# Atherosclerosis and Chronic Apical Periodontitis: Systematic Review and Meta-Analysis

**DOI:** 10.3390/jcm14051504

**Published:** 2025-02-24

**Authors:** María León-López, Daniel Cabanillas-Balsera, Jenifer Martín-González, Benito Sánchez-Domínguez, Juan J. Saúco-Márquez, Juan J. Segura-Egea

**Affiliations:** 1 Endodontic Section, Department of Stomatology, School of Dentistry, University of Sevilla, C/Avicena s/n, 41009 Sevilla, Spain; maria.leon.lopez.98@gmail.com (M.L.-L.); dcabanillas@us.es (D.C.-B.); jmartin30@us.es (J.M.-G.); jjsauco@us.es (J.J.S.-M.); 2 Andalusian Health Service, Distrito Sanitario Sevilla, 41013 Sevilla, Spain; beni2506@yahoo.es

**Keywords:** apical periodontitis, atherosclerosis, endodontics, epidemiology, population-based study, prevalence, systematic review, survey

## Abstract

**Background:** Atherosclerosis is a chronic and progressive condition of the arteries, characterized by the thickening and hardening of their walls due to the formation of atherosclerotic plaques. Low-grade inflammation is implicated in the pathogeny of atherosclerosis. Chronic apical periodontitis (CAP), the chronic inflammation around the root apex of infected teeth, is associated with a low-grade inflammatory state, and thus a connection between atherosclerosis and CAP has been suggested. The aim of this study was to conduct a systematic review with meta-analysis to answer the following PICO question: In adult patients, does the presence or absence of atherosclerosis affect the prevalence of CAP? **Methods:** The PRISMA guidelines were followed to carry out this systematic review, which was registered in PROSPERO (651359). A bibliographic search was performed in PubMed-MEDLINE, Embase, and Scielo. The inclusion criteria selected studies presenting data on the prevalence of CAP in patients diagnosed with atherosclerosis and control patients. The statistical analysis was carried out using RevMan software v.5.4. The study characteristics and risk ratios with 95% confidence intervals (CIs) were extracted. Random-effects meta-analyses were performed. Risk of bias was assessed using the Newcastle-Ottawa scale, which was adapted for cross-sectional studies. To estimate variance and heterogeneity between trials, the Higgins I2 test was used. The quality of the evidence was evaluated using GRADE. **Results**: The search strategy recovered 102 articles, and only five met the inclusion criteria. Meta-analysis showed an overall OR = 2.94 (95% CI = 1.83–4.74; *p* < 0.01) for the prevalence of CAP among patients with atherosclerosis. The overall risk of bias was moderate. The quality of the evidence showed a low level of certainty. **Conclusions:** Patients with atherosclerosis are almost three times more likely to have CAP. This finding supports the hypothesis that chronic inflammatory processes in the oral cavity could significantly impact cardiovascular health, emphasizing the importance of an integrated approach to oral and systemic health care. This result should be translated to daily clinical practice, and the healthcare community should be aware of this association and suspect atherosclerotic pathology in patients who show a high prevalence of CAP. Likewise, patients with atherosclerosis should be monitored in the dental clinic for CAP.

## 1. Introduction

Apical periodontitis is an inflammation of the tissues surrounding the apex of the tooth caused by an immune reaction triggered by bacterial antigens passing from the infected root canal [1]. The estimated prevalence of apical periodontitis is about 52% [2]. Periapical chronic inflammation, called chronic apical periodontitis (CAP), causes destruction of the periapical bone, which is evident in X-rays as a radiolucent image around the apex of the affected tooth [3].

Atherosclerosis is a chronic and progressive condition of the arteries characterized by the thickening and hardening of their walls due to the most common formation of atheromatous plaques [4]. These plaques are accumulations of lipids, cholesterol, calcium, and other substances that area deposited in the innermost layer of the arteries, known as the intima, which is in contact with the blood and lined with endothelial cells [5]. Atheromatous plaques can restrict blood flow or even completely obstruct an artery, leading to severe cardiovascular problems such as myocardial infarctions or strokes [4].

Atherosclerosis can be characterized as an independent form of inflammation, sharing similarities with but also having fundamental differences from low-grade inflammation and various variants of canonical inflammation [6]. There are numerous factors which act as inducers of the inflammatory process in atherosclerosis, including vascular endothelium aging, metabolic dysfunctions, and autoimmune and, in some cases, infectious damage factors [4,6]. Low-grade chronic inflammation can lead to vascular inflammation and endothelial dysfunction, which can subsequently lead to atherosclerosis [6,7]. Oral inflammatory processes, such as periodontal disease [8] and chronic apical periodontitis [9], are associated with low-grade inflammation [10]. For this reason, studies have been conducted investigating the possible association of periodontal disease [11] and chronic apical periodontitis [12] with atherosclerosis. Bacteremia, with the spreading of infection from the periapical tissues to atherosclerotic plaque, could be a link between CAP and atherosclerosis. CVD-associated taxa such as *Porphyromonas gingivalis* and *Fusobacterium nucleatum*, which are involved in endodontic infection, may contribute to atherosclerosis and endothelial dysfunction [13].

Early endothelial dysfunction has been found in young men with CAP compared with healthy control patients [14]. Some studies have found a relationship between CAP and cardiovascular diseases associated with atherosclerosis, such as myocardial infarction [15], hypertension [16], or stroke [17]. Additionally, some experimental studies carried out in animals have found that CAP exacerbates atherosclerosis [18,19]. Moreover, CAP shares common risk factors with diseases associated with atherosclerosis. Some genetic polymorphisms increase susceptibility to chronic inflammation, modulating systemic pro-inflammatory mediators, which can favor both CAP and atherosclerosis [20,21]. Likewise, both CAP and atherosclerosis share environmental risk factors, such as diabetes and smoking, which in addition to contributing to inflammatory susceptibility have a direct influence at the periapical and cardiovascular levels [22,23].

This study aimed to conduct a systematic review with meta-analysis to investigate the possible association between atherosclerosis and the prevalence of CAP. The null hypothesis was that the prevalence of CAP in patients with atherosclerosis is similar to that of the healthy general population.

## 2. Materials and Methods

To conduct this systematic review, the Preferred Reporting Items for Systematic Reviews and Meta-Analyses (PRISMA) guidelines [24,25] were followed. This systematic review has been submitted to PROSPERO (651359).

### 2.1. Review Question

The review focused on answering the following research question, structured according to the population, intervention, comparison, and outcome (PICO) method: In adult patients (P), does the presence of atherosclerosis (I), compared with its absence (C), affect the prevalence of CAP?

### 2.2. Eligibility Criteria

According to the research question, the following selection criteria were established.

#### 2.2.1. Inclusion Criteria

The inclusion criteria established were (1) epidemiological studies published until December 2024; (2) studies comparing atherosclerotic patients with healthy control subjects; (3) studies diagnosing the atherosclerotic condition clinically or via imaging techniques; and (4) studies assessing the prevalence of AP, both in atherosclerotic patients and in control healthy subjects, using radiological methods.

#### 2.2.2. Exclusion Criteria

The exclusion criteria were as follows: (1) in vitro or animal studies; (2) case series; (3) studies reporting data only from atherosclerotic patients; (4) studies not reporting the radiological prevalence of CAP; (5) studies including patients with atherosclerosis in the same experimental group as patients with other pathologies, without differentiating the data obtained in each pathology; and (6) studies which had no initial agreement among the reviewers.

### 2.3. Search Strategy and Information Sources

Once the PICO question and inclusion criteria were established, the search strategy was designed. A bibliographic search was conducted with no time or language limits in PubMed/MEDLINE, Web of Science, Scopus, EMBASE, and SCIELO. For the electronic search strategy, terms from the Medical Subject Heading (MeSH) and text words (tw) were combined as follows: (“arteriosclerosis”[All Fields] OR “coronary artery disease”[All Fields] OR “atherosclerosis”[All Fields] OR “arterial disease”[All Fields] OR “atherosclerotic plaque”[All Fields]) AND (“periapical pathology”[All Fields] OR “apical periodontitis”[All Fields] OR “periapical infection”[All Fields] OR “endodontics”[All Fields] OR “periapical disease”[All Fields]). A complementary screening was performed by searching for any additional studies through the references of the included studies which did not appear in the database search. A search was conducted in the gray literature, but it did not provide useful data (URL: https://opengrey.eu/; accessed on 13 November 2024; URL: https://scholar.google.com/; accessed on 13 November 2024; and URL: https://www.greynet.org/, accessed on 13 November 2024).

The selection of the articles was performed by three authors individually (J.J.S.-E., M.L.-L., and D.C.-B.) through reading the titles and abstracts or the full text if further clarification was necessary. A second stage involved reading the full texts of all selected articles to definitively assess whether or not they met the inclusion criteria. Disagreements on eligibility were resolved by discussion and consensus.

### 2.4. Data Extraction

One of the authors (M.L.-L.) was responsible for data extraction, while three reviewers (J.M.-G., D.C.-B., and J.J.S.-E.) verified the tabulated data to ensure the absence of typo errors and carried out the analysis of the articles. Any articles in disagreement were discussed. For each study, the following data were extracted: authors and year of publication, study design, the total sample size, the size of the control and experimental groups, main variables, methods used to diagnose CAP and detect atherosclerosis, and the main results, including the number of control and atherosclerotic patients with and without CAP.

### 2.5. Data Synthesis and Analysis

The outcome variable studied was the prevalence of CAP, calculated as the percentage of individuals with at least one tooth affected by CAP. In each selected study, the odds ratio (OR) was calculated with its 95% confidence interval (CI), aiming to measure the effect of the relationship between atherosclerosis and the prevalence of CAP.

To determine the overall OR and its 95% CI for the prevalence of AP, a random-effects meta-analysis was performed using RevMan software (Review Manager Web. The Cochrane Collaboration, 2019) version 5.4. To estimate the variance and heterogeneity of the studies, the Tau^2^ and Higgins I^2^ tests were used, considering slight heterogeneity if it ranged from 25% to 50%, moderate heterogeneity from 50% to 75%, and high heterogeneity if it was greater than 75% [26]. The level of significance was applied to *p* = 0.05.

### 2.6. Risk of Bias Assessment

The risk of bias of each study was evaluated with the Newcastle-Ottawa Scale [27], modified for cross-sectional studies [28]. The scale was modified according to the outcome of interest, and the subjects were categorized into two domains: sample selection and outcome. Each aspect was evaluated by assigning points (*) depending on whether it was present or not. Three authors (M.L-L., D.C-B., and J.J.S-E.) independently assessed the risk of bias of each of the included studies. The authors discussed them until reaching a consensus. Two domains were established: one related to how the sample was selected and another related to the outcome. The following criteria were used to evaluate each section:

(A) Domain “Sample selection” (maximum = six points):
  (1) Representativeness of the sample: Random sampling ➔ three points; non-random sampling ➔ two points; elected group of patients ➔ one point; no explanation of the sampling plan ➔ no points.
  (2) Sample size: The method for sample size calculation is provided, or the entire population was enlisted (with loss rate ≤ 20%) ➔ one point; size calculation not provided ➔ no points.
  (3) Atherosclerotic condition: The atherosclerotic condition was verified with imaging techniques ➔ two points; atherosclerotic condition was established only clinically ➔ one point; the condition of atherosclerosis was not established either clinically or with imaging methods ➔ no points.
(B) Domain “Outcome” (maximum = six points):
  (1) Evaluation of the outcome: CAP was diagnosed by a trained and calibrated observer, providing inter- and intra-agreement values ➔ two points; CAP was diagnosed by trained and calibrated observers, without providing inter- and intra-agreement values ➔ one point; CAP was diagnosed without specifying training or calibration of the observers ➔ no points.
  (2) Type of radiographs used: Computed tomography or periapical radiographs ➔ two points; orthopantomography ➔ one point; the type of X-ray used was not mentioned ➔ no points.
  (3) Third molars are assessed: Yes ➔ one point; no ➔ no points.
  (4) Number of observers: Two or more ➔ one point; only one ➔ no points.

The lowest possible risk of bias was scored with 12 points. Studies with scores from 0 to 4 points were considered to be at high risk of bias, those with scores between 5 and 8 points were considered to be at moderate risk of bias, and finally, studies with scores between 9 and 12 points were considered to be at low risk of bias.

Articles were assessed independently by 4 reviewers (J.J.S.-E., D.C.-B., M.L.-L., and J.J.S.-M.), and cases of disagreements over the risk of bias were discussed until a consensus was achieved.

### 2.7. Grading Recommendations Assessment, Development, and Evaluation

The Grading Recommendations Assessment, Development and Evaluation (GRADE) tool was used to assess the overall quality and certainty of the evidence [29]. Four investigators (M.L.-L. J.J.S.-E., B.S.-D., and J.J.S.-M.) independently carried out the assessment. This procedure defines an initial level of certainty according to the design of the included studies and subsequently analyzes different domains, such as the risk of bias, inconsistency, indirectness, imprecision, publication bias, dose-response gradient, confounding factors, or the magnitude of the effect, concluding with a final level of certainty. Having a high or moderate level of evidence, whether it was highly likely or possible that the true effect was close to the estimated conclusion, would allowed a recommendation to be made. A low or extremely low level of evidence indicates that confidence in the result is limited or extremely weak, respectively [30].

## 3. Results

**Figure 1:**
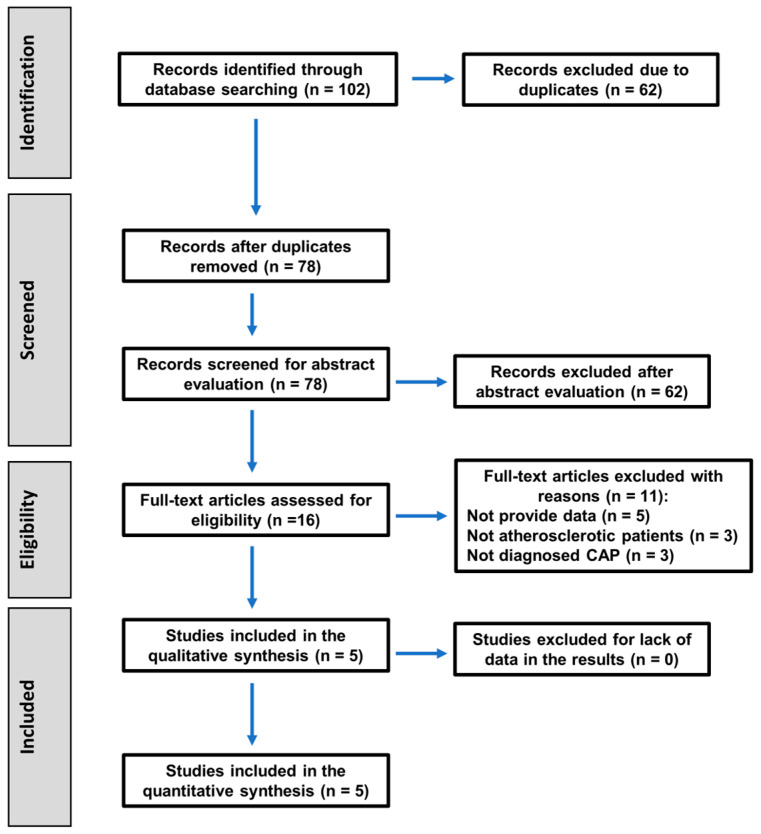
Flowchart of the search strategy following the PRISMA 2020 guidelines for systematic reviews and meta-analyses.

The search strategy to select the articles included in this systematic review followed the PRISMA 2020 guidelines, as shown in the flowchart in Figure 1.

The baseline search recovered 102 titles. After the removal of duplicates, 78 articles remained, of which 62 were excluded after reading their abstracts for being unrelated to the topic, and 16 were selected for their full text. After full-text reading, 11 articles were excluded for the following reasons. Five did not provide the necessary data [12,14,31,32,33]. In three studies, some patients in the experimental group did not have atherosclerosis [34,35,36], and the other three studies did not establish a radiographic diagnosis of CAP [37,38,39]. The excluded articles and the reasons for their exclusion are shown in Table 1.

### 3.1. Characteristics of the Included Studies

Finally, five studies were selected and included for systematic review and meta-analysis (Table 2).

The studies [15,40,41,42,43] analyzed the association between atherosclerosis and the prevalence of CAP, which was diagnosed radiographically. For each study, the study design, included subjects, variables analyzed, diagnostic methods, and main results are summarized in Table 2. Four of the included studies were cross-sectional [40,41,42,43], with level 4 evidence according to the Oxford Centre for Evidence-Based Medicine [44], and one was a retrospective study [15] with level 3 evidence. Regarding the diagnosis of CAP, three of the included studies used panoramic radiographs to identify teeth with apical pathology [15,42,43], one study used periapical radiographs [41], and another study used a whole-body CT scan [40] (Table 2).

Concerning the atherosclerotic condition of patients in the atherosclerosis group, two studies included patients who had suffered cardiovascular events (CVEs) associated with atherosclerosis [15,42]. Another study used a CT scan [40], another one used coronary angiography [41], and the fifth one used Doppler sound to assess the carotid wall’s thickness [43].

An evidence table was created with data from the included studies (Table 3). The number of patients with at least one tooth with CAP in both the control and atherosclerotic groups were the key data extracted in each of the studies. Then, the prevalence of CAP in the patients with atherosclerosis and the healthy control subjects was calculated, as well as the corresponding OR for each study and the overall sample.

The five studies included a total of 1108 people: 515 atherosclerotic patients and 593 control healthy subjects. The patients with atherosclerosis showed a CAP prevalence of 65.4%, which was much higher than that found in the control group (38.8%). Data from four of the five studies [40,41,42,43] provided significant OR values greater than one (1.65–3.09), indicating association between atherosclerosis and periapical status. One of the studies [15] did not provide a significant OR (1.65; *p* = 0.126). The calculated overall OR was OR = 2.99 (95% CI = 2.34–3.82; *p* = < 0.001).

### 3.2. Meta-Analysis of the Prevalence of Chronic Apical Periodontitis

**Figure 2:**
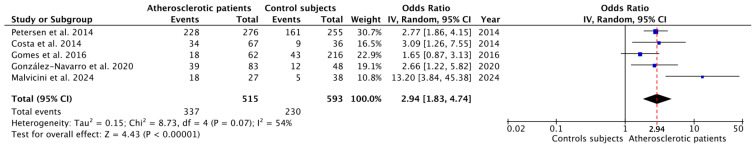
Forest plot of the OR and its 95% confidence interval comparing the prevalence of apical periodontitis in patients with atherosclerosis and healthy control patients. The estimate was based on data from the five selected studies.

The data shown in Table 3 were used to perform the meta-analysis, the forest plot of which is shown in Figure 2.

The blue squares represent the point estimate of the OR, and these are proportional in area to the study size. The horizontal lines represent the 95% confidence intervals. The black diamond shows the statistical summary of the five studies, and the red dotted vertical line indicates the calculated overall OR. This overall OR was determined using the inverse variance random effects method, resulting in an OR = 2.94 (95% CI = 1.83–4.74; *p* < 0.01). The heterogeneity value was I^2^ = 54% (Tau^2^ = 0.15; *p* = 0.07), indicating a moderate level of heterogeneity among the studies.

### 3.3. Risk of Bias Assessment

According to the Newcastle-Ottawa Scale [27], risk of bias was evaluated for each study (Table 4). The five studies were classified as having a moderate risk of bias. The overall sum of the scores of the five studies was 30, indicating a moderate overall risk of bias.

### 3.4. Publication Bias

**Figure 3:**
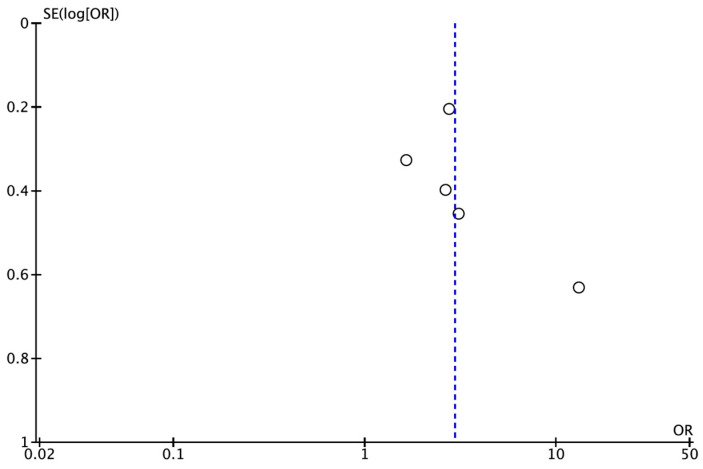
Funnel plot for estimates in the meta-analysis of the prevalence of CAP in patients with atherosclerosis and healthy control patients. Studies with higher power levels and lower standard error are placed toward the top. Studies with lower power levels are placed toward the bottom.

Publication bias could not be assessed quantitatively as there were fewer than the required minimum of 10 studies [26]. However, a funnel plot was plotted to illustrate the possible existence of publication bias (Figure 3).

### 3.5. GRADE Evaluation: Level of Certainty

The certainty of evidence was assessed using the GRADE tool (Table 5). All articles reported observational studies; therefore, the initial level of certainty was low. The risk of bias domain, according to its overall result (moderate), was classified as “not serious”. The inconsistency domain was considered “not serious” as the heterogeneity was moderate (I^2^ = 54%).

Taking into account that the included studies did not perform indirect comparisons or present indirect results (i.e., the included populations were representative of the atherosclerotic patients, and CAP was reliably evaluated), the indirectness domain was scored as “not serious”. However, the domain of imprecision was rated “serious” since the 95% CI of the estimated effect (OR) was out of the 0.75–1.25 range, and the number of included studies was moderate (only five studies).

Publication bias could not be assessed quantitatively due to the low number of studies, and the funnel plot did not indicate that it was sufficient to downgrade the quality of the evidence. In fact, studies with variable sample sizes and not funded by the private sector were included in this systematic review. Consequently, the certainty of the evidence was considered low, indicating that the overall OR obtained could differ widely from the real one.

## 4. Discussion

The aim of this systematic review and meta-analysis was to investigate the relationship between the prevalence of CAP and atherosclerosis. After analyzing the studies published in the literature to date, the null hypothesis can be rejected; that is, the prevalence of CAP was higher in the patients with atherosclerosis compared with the healthy control subjects. This finding supports the hypothesis that CAP could significantly impact cardiovascular health, emphasizing the importance of an integrated approach to oral and systemic healthcare.

The overall OR provided by the meta-analysis (OR = 2.94; *p* < 0.01) suggests that patients with atherosclerosis are nearly three times more likely to present CAP than patients without this condition. However, the overall risk of bias was moderate, and the evaluation of the level of certainty, carried out with the GRADE tool [30], concluded that the level of certainty was low; that is, the true effect might be markedly different from the estimated effect.

### 4.1. Methodological Differences and Heterogeneity

Among the included studies, there are marked differences in the methodologies used to detect apical periodontitis and to diagnose atherosclerosis. This probably influenced the calculated heterogeneity value (I^2^ = 54%). Each radiographic technique has its own limitations for diagnosing periapical pathology. Periapical and panoramic radiographs are useful for initial evaluations of pathology [45], but CBCT provides more advanced and detailed information [46]. However, CBCT has significant drawbacks, such as its high cost and lower accessibility, and thus it should be reserved for specific cases of diagnosis and follow-up of periapical pathology [47].

Regarding the diagnosis of atherosclerotic conditions, all included studies confirmed the atherosclerotic status of patients clinically or through imaging techniques. Therefore, there was correspondence between the conditions of the included studies and the specific research PICO question without indirectness, and this was considered in the evaluation of the level of certainty.

The first study investigating the possible association between CAP and coronary heart disease was carried out in 2003 [36], but it was not included in this review because the study sample involved patients with different pathologies. On the contrary, all of the studies included in the systematic review determined the relationship between the prevalence of CAP in control healthy subjects and in patients with atherosclerotic conditions, assessed clinically or by imaging techniques.

The study by Petersen et al. (2014) [40] was the one which contributed the highest percentage of subjects (48%) to the meta-analysis and also the one which found the highest prevalence of CAP in both atherosclerotic patients and controls. The results of this study provided an OR of 2.83 (*p* < 0.01) for the association between atherosclerosis and CAP. Three other studies [41,42,43] included in the systematic review also provided similar OR values. On the contrary, the study conducted by Gomes et al. (2016) [15] including 278 subjects provided a non-significant result of OR = 1.65 (*p* = 0.126). The prevalence of CAP found both in the control group (20%) and the atherosclerotic group (29%) in this study was strikingly low compared with that calculated for the healthy world population (52%) [2]. This could explain its non-significant result. Other differences in the results of the included studies may be attributed to variability in the methodologies used to diagnose CAP and atherosclerosis, as well as population and design differences.

### 4.2. Pathophysiological Implications

Although it was not the objective of this study, the application of the criteria of biological plausibility and scientific coherence stated by Hill [48] advises analyzing the possible biological mechanisms which could explain the association between endodontic infection and atherosclerosis. The relationship between CAP and atherosclerosis can be explained by several pathophysiological mechanisms [22]. Chronic inflammation from CAP may induce endothelial dysfunction [14,49], a critical step in the development of atherosclerosis. Endothelial dysfunction, characterized by reduced nitric oxide production, adversely affects vascular tone regulation and promotes the formation of atherosclerotic plaques [49].

Low-grade chronic inflammation can also increase the risk of atherosclerosis and insulin resistance, which are the leading mechanisms in the development of cardiovascular diseases (CVDs) [50]. Apical periodontitis is associated with elevated levels of inflammatory markers such as interleukins, immunoglobulins, and C-reactive protein in humans [51]. These findings suggest that apical periodontitis contributes to low-level systemic inflammation [52] and is not limited to a localized lesion, leading to an increased risk of cardiovascular diseases [53]. Additionally, inflammatory mediators released during CAP, such as interleukins and C-reactive protein, may enter the bloodstream, contributing to vascular damage [8,54,55]. The presence of chronic inflammation in adults with CAP could act as a risk factor for various cardiovascular diseases, contributing to a reduction in flow-mediated dilation and an increase in carotid intima thickness [14,43].

Another proposed mechanism involves the potential role of bacterial dissemination. Pathogens associated with CAP, such as *Porphyromonas endodontalis* and *Enterococcus faecalis*, may invade the bloodstream and adhere to vascular endothelium, directly contributing to plaque formation and vascular inflammation [22,56,57]. This bacterial insult can synergize with systemic inflammation to accelerate the progression of atherosclerotic lesions, highlighting the multifaceted nature of this association [58].

Experimental studies have also demonstrated that CAP can exacerbate atherosclerosis. Conti et al. (2020) [18] and Gan et al. (2023) [59] documented that animal models with CAP developed higher levels of systemic inflammation and atherosclerotic plaque formation compared with their controls. These findings support the hypothesis of a bidirectional connection between oral inflammatory pathologies and cardiovascular diseases [60].

### 4.3. Clinical Implications

The results of this systematic review and meta-analysis have an evident clinical interest, as they suggest a relationship between atherosclerosis and endodontic disease. Dentists should be aware that CAP could serve as a potential cardiovascular risk indicator, and physicians should consider oral health as an integral part of the evaluation and management of patients with atherosclerosis. Interdisciplinary collaboration is essential to address these risks effectively. For example, early detection and treatment of CAP could reduce systemic inflammatory states and potentially mitigate the progression of atherosclerosis. Similarly, periodic monitoring of atherosclerosis patients in dental clinics could help identify undiagnosed CAP cases. Therefore, it is essential to consider this relationship in the daily clinical practice of dentists and healthcare professionals in general.

This systematic review, together with other published studies [61,62,63,64], highlights the relationship between CAP and several systemic diseases [22,60], making it crucial for clinicians to study these relationships to better understand and communicate the prognosis of a tooth undergoing endodontic treatment. Moreover, systemic diseases can influence the outcome of root canal treatment [54].

However, the results of this review must be considered in their entirety. The GRADE system was applied to assess the certainty of evidence, starting at a low level due to the observational nature of the included studies. Domains such as risk of bias, inconsistency, and indirectness were classified as “not serious”, while imprecision was rated “serious” due to the confidence intervals and the limited number of studies. Overall, the evidence was classified as low certainty, suggesting that the true effect might differ substantially from the estimated effect.

### 4.4. Strengths and Limitations of This Study

The conducted meta-analysis was based on a robust methodological approach, adhering to the PRISMA guidelines for systematic reviews [24]. The inclusion of five studies involving 1108 participants allowed for a statistical synthesis providing a highly significant OR for the association between atherosclerosis and CAP. The use of standardized tools, such as the Newcastle-Ottawa Scale and the GRADE system, ensured a rigorous evaluation of bias and evidence quality.

Despite its strengths, this study has notable limitations. First, the number of studies included in the meta-analysis was limited (n = 5), which may have affected the generalizability of the results. Additionally, methodological heterogeneity among the studies was moderate (I^2^ = 54.2%), reflecting differences in the diagnostic techniques used to identify CAP and atherosclerosis. For instance, while some studies employed panoramic radiographs, others used periapical radiographs or computed tomography, potentially influencing the detection of periapical lesions.

Another limitation is the lack of population representativeness in the samples studied. Most patients were selected from university dental clinics, potentially introducing selection bias. Furthermore, not all studies excluded edentulous patients, which might have altered the CAP prevalence estimates.

### 4.5. Future Directions

Future studies should focus on prospective designs to establish causal relationships between CAP and atherosclerosis. Additionally, evaluating the impact of endodontic treatment on the progression of atherosclerosis and exploring the role of specific inflammatory biomarkers in this association would be valuable. Finally, the inclusion of more diverse and representative samples will allow for greater generalizability of results.

## 5. Conclusions

This systematic review and the meta-analysis suggest a significant association between atherosclerosis and CAP. Patients with atherosclerosis are almost three times more likely to have CAP, highlighting the importance of considering oral health as a key component in the prevention and management of cardiovascular diseases. This result should be translated to daily clinical practice. Multidisciplinary collaboration is essential to integrate oral care strategies into medical practice and improving the overall health outcomes in patients with these chronic conditions. It is important for the healthcare community to be aware of this association in order to suspect atherosclerotic pathology in patients who show a high incidence of periapical pathology in their dental history.

## Figures and Tables

**Table 1:** Excluded studies and their reasons for exclusion.

Reasons	Excluded Studies
Not providing the necessary data	Friedlander et al., 2010 [31] Cotti et al., 2011 [9] Glodny et al., 2013 [32] Liljestrand et al., 2016 [33] Chauhan et al., 2019 [12]
Some patients in the experimental group did not have atherosclerosis	Frisk et al., 2003 [36] Willershausen et al., 2009 [34] Pasqualini et al., 2012 [35]
Not establishing a radiological diagnosis of CAP	Jansson et al., 2001 [37] Cowan et al., 2020 [38] Liu et al., 2023 [39]

**Table 2:** Studies on the prevalence of CAP and atherosclerosis included in the meta-analysis: study design, subjects and sample size, diagnostic methods, and main results.

Author, Year	Study Design	Subjects	Diagnostic Methods for CAP and Atherosclerotic Condition	Main Results
Petersen et al., 2014 [40]	Cross-sectional	Control: 255 Atherosclerotic: 276	CT scan Healing scanning from CTs of abdominal aorta	CAP correlated positively with the aortic atherosclerotic burden
Costa et al., 2014 [41]	Cross-sectional	Control: 36 Atherosclerotic: 67	Periapical radiographs Coronary angiography	CAP was independently associated with coronary artery disease
Gomes et al., 2016 [15]	Retrospective	Control: 216 Atherosclerotic: 62	Ortopantomography Incident CVE	The number of teeth with CAP in midlife was an independent predictor of CVE
González-Navarro et al., 2020 [42]	Cross-sectional	Control: 48 Atherosclerotic: 83	Ortopantomography Having suffered an atherotrombotic CVE	CAP was significantly associated with atherotrombotic CVE
Malvicini et al., 2024 [43]	Cross-sectional	Control: 38 Atherosclerotic: 27	Ortopantomography Carotid wall thickness Doppler ultrasound	CAP was associated with fivefold increased odds of having carotid plaques

**Table 3:** Studies about atherosclerotic patients and the prevalence of CAP. Results extracted and compiled, with descriptive statistics and odds ratios calculated.

Authors, Year	No. Subjects	Atherosclerotic Patients		Control Subjects		Odds Ratio (95%CI)	*p*
		Cap/Total	CAP Prevalence (%)	CAP/Total	CAP Prevalence (%)		
Petersen et al., 2014 [40]	531	228/276	82.6	161/255	63.1	2.77 (1.86–4.15)	<0.001
Costa et al., 2014 [41]	103	34/67	50.7	9/36	25.0	3.09 (1.26–7.55)	0.012
Gomes et al., 2016 [15]	278	18/62	29.0	43/216	19.9	1.65 (0.87–3.13)	0.126
González-Navarro et al., 2020 [42]	131	39/83	47.0	12/48	25.0	2.66 (1.22–5.82)	0.013
Malvicini et al., 2024 [43]	65	18/27	66.7	5/38	13.2	13.20 (3.84–45.38)	< 0.001
OVERALL	1108	337/515	65.4	230/593	38.8		

CAP: chronic apical periodontitis.

**Table 4:** Risk of bias of individual studies assessed using the Newcastle-Ottawa Scale. The maximum possible score was 12 points (60 points for the five studies). High risk of bias was defined as 0–4 points, moderate risk of bias was considered for the studies scoring 5–8 points, and finally low risk of bias was assigned to studies scoring between 9 and 12 points. Each * is one point.

	Sample Selection			Outcome				Risk of Bias
	Representativeness of the Sample (Max 3)	Sample Size Calculation (Max 1)	Atherosclerotic Condition (Max 2)	Outcome Assessment (Max 2)	Type of Radiograph (Max 2)	Inclusion of Third Molar (Max 1)	No. of Observers (Max 1)	
Petersen et al., 2014 [40]			**		**		*	5 (moderate)
Costa et al., 2014 [41]	*	*	**		**		*	7 (moderate)
Gomes et al., 2016 [15]			*	**	*		*	5 (moderate)
González-Navarro et al., 2020 [42]	*		*		*	**	*	6 (moderate)
Malvacini et al., 2024 [43]	*		**		*	**	*	7 (moderate)
OVERALL	3	1	8	2	7	4	5	30 (moderate)

**Table 5:** Grade assessment of certainty level (GRADE Working Group) [23].

Certainty Assessment							Certainty	Importance
No. of Studies	Study Design	Risk of Bias	Inconsistency	Indirectness	Imprecision	Other Considerations		
Atherosclerosis—apical periodontitis								
5	Observational studies	Not serious ^a^	Not serious ^b^	Not serious	Serious ^c^	OR: 2.94 (1.83–4.74) *p* < 0.01	Low	IMPORTANT

Explanations: ^a^ Detailed in Table 4: Risk of bias summary (moderate). ^b^ I^2^ = 54% (*p* = 0.07). ^c^ 95% CI outside of 0.75–1.25. Low certainty: The true effect might be markedly different from the estimated effect.

## Data Availability

The data are public.

## Abbreviations

The following abbreviations are used in this manuscript:

CAP	Chronic apical periodontitis
CI	Confidence interval
CT	Computed tomography
OR	Odds ratio
